# Deep Retraction Pockets, Endometriosis, and Quality of Life

**DOI:** 10.3389/fpubh.2016.00085

**Published:** 2016-05-09

**Authors:** Patrick P. Yeung, Ian Logan, Jeffrey A. Gavard

**Affiliations:** ^1^Division of Gynecologic Surgical Subspecialties, Department of Obstetrics, Gynecology, and Women’s Health, Center for Endometriosis, Saint Louis University, St. Louis, MO, USA; ^2^School of Medicine, Saint Louis University, St. Louis, MO, USA; ^3^Department of Obstetrics, Gynecology, and Women’s Health, Saint Louis University, St. Louis, MO, USA

**Keywords:** deep dyspareunia, quality of life, endometriosis, excision surgery, peritoneal window, retraction pocket, Allen-Masterson window, atypical endometriosis

## Abstract

**Objective:**

The purpose of this study was to examine if deep retraction pockets (DRPs) in the posterior cul-de-sac and uterosacral ligaments could be a manifestation of endometriosis and if excision of these pockets improves pain symptoms and quality of life.

**Study design:**

Prospective cohort study Canadian Task Force Classification, II-3.

**Materials and methods:**

Preoperative data, operative data, and follow-up data were collected prospectively at the Center for Endometriosis at Saint Louis University, a referral center for the surgical management of endometriosis.

**Results:**

The 107 consecutive patients who presented with preoperative deep dyspareunia were included in the study, and the median postoperative follow-up was 13 months. Endometriosis was confirmed histologically in any location excised in 88/107 (82.2%) of the women, and 31 DRPs were excised from 25 women with DRPs in the posterior cul-de-sac or uterosacral ligaments, of which 15/31 (48.4%) had endometriosis. Of the 10 DRPs without visible surface lesions, 3 (30.0%) had endometriosis on histology. Pain symptoms and quality of life significantly improved after excision surgery, whether or not DRPs were present. Women who had endometriosis in their DRP also had significant improvement in deep dyspareunia and chronic pelvic pain and quality of life. Results did not differ when patients who took postoperative hormonal suppression were removed from the analyses.

**Conclusion:**

Patients had significantly improved pain symptoms and quality of life after excision surgery, whether or not DRPs were present. This study demonstrated that a DRP may be a manifestation of endometriosis (even with a clear surface of the pocket), so that DRPs should be excised to achieve optimal excision of endometriosis.

## Introduction

**Figure 1:**
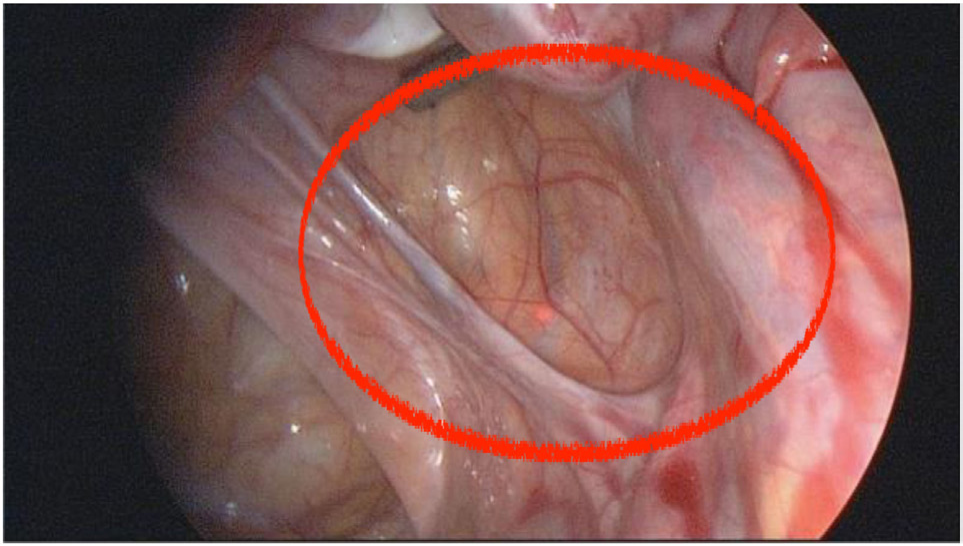
**This annotated photo shows the appearance of a deep retraction pocket, with a visible manifestation of endometriosis, seen at laparoscopy for pelvic pain**. There are abnormal red and white lesions suspicious for endometriosis at the base of the pocket.

A pelvic peritoneal pocket (or Allen-Masterson pocket) in association with endometriosis (Figure 1) was first described by Sampson (1). Chatman (2), Chatman and Zbella (3), and Redwine (4) later demonstrated the importance of recognizing peritoneal pockets as a potential manifestation of endometriosis, as endometriosis in such structures in women with pelvic pain otherwise could remain undiagnosed and untreated.

Vercellini et al. (5) showed a poor correlation between location of pain and location of superficial disease. Hsu et al. (6) showed a stronger correlation between location of pain and location of deep disease.

The purpose of this study was to examine if deep retraction pockets (DRPs) in the posterior cul-de-sac and uterosacral ligaments could be a manifestation of endometriosis, and if excision of these pockets improves pain symptoms and quality of life.

### Study Design

Prospective cohort study Canadian Task Force Classification, II-3.

## Materials and Methods

Consecutive patients seen for pelvic pain at the Center for Endometriosis at Saint Louis University (SLU), a referral center for the surgical management of endometriosis, were recruited for the study. Patients included in this study were those who described deep dyspareunia on a standard of care preoperative questionnaire and who underwent laparoscopic excision surgery to relieve pain symptoms. All patients underwent optimal or complete excision of all visible manifestations of endometriosis, including peritoneal retraction pockets.

Preoperative data, operative data, and follow-up data were collected prospectively as part of an on-going database approved by the SLU Institutional Review Board (IRB). Symptom data were collected on deep dyspareunia and dyschezia by a 10-point visual analog scale (VAS) and on quality of life by a simple 100-point scale (7) before and after excision surgery. Operative data collected included location of abnormal peritoneum and DRPs (defined as estimated to be greater than 0.5 cm), phenotype or color of lesions excised, and diagnosis of endometriosis by histology. Patients were not specifically recommended to take hormonal suppression after surgery, though they were free to do so for contraception or to induce amenorrhea.

Differences in demographic characteristics, surgical characteristics, baseline severe pain symptoms (≥7 versus <7), and baseline quality of life (≥70 versus <70) between women with and without DRP were assessed using chi-square test and Fisher’s Exact test for categorical variables. Independent Student’s *t*-test or the Kolmogorov–Smirnov test was used for continuous variables depending on the normality of the distributions. Changes in pain symptoms and quality of life from before surgery to after surgery for women with DRP and women without DRP were assessed using paired *t*-tests or the Wilcoxon signed-rank test depending on distribution normality. Preoperative, postoperative, and rate of change of pain scores, and quality of life between women with and without DRP were compared using the independent Student’s *t*-test or the Kolmogorov–Smirnov test. A *p*-value of <0.05 was used to denote statistical significance. All analyses were performed using SPSS version 23.0 for Windows.

## Results

**Figure 2:**
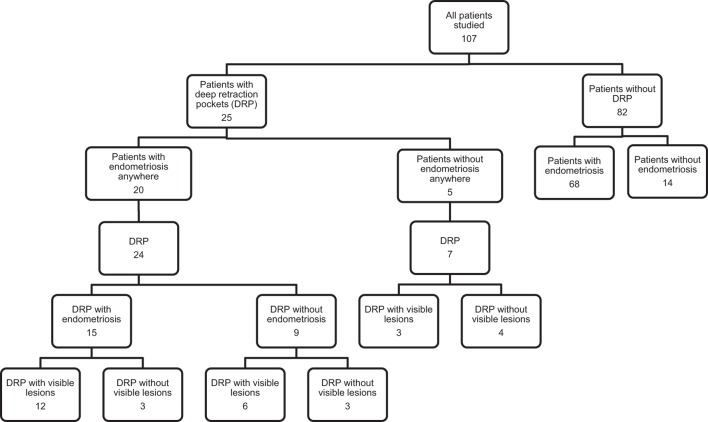
**Flow of participants through the study**.

The 107 consecutive patients who presented with preoperative deep dyspareunia from February 20, 2012 to July 16, 2013 were included in the study (Table 1; Figure 2). The postoperative questionnaire was completed by 53/107 (49.5%) of the women at a median time after surgery of 13 months and a range of 12–25 months.

**Table 1:** **Deep retraction pockets in women with deep dyspareunia**.

Sample studied	Numerator/denominator	%
**Total patients**	**107**	
Patients with endometriosis anywhere	88/107	82.2
Patients with DRP	25/107	23.4
Patients with endometriosis in a DRP	15/25	60.0
**Total deep retraction pockets**	**31**	
DRP with endometriosis	15/31	48.4
DRP that were clear (no visible lesions)	10/31	32.3
Clear DRP with endometriosis	3/10	30.0

*DRP, deep retraction pocket*.

*Compare with Figure 2*.

Endometriosis was confirmed histologically in any location excised in 88/107 (82.2%) of the women (Table 1; Figure 2). Twenty-five of the 107 women (23.4%) had DRP in the posterior cul-de-sac or uterosacral ligaments, of whom 15/25 (60.0%) had endometriosis confirmed in at least one DRP. Of the women with confirmed endometriosis anywhere, 20/88 (22.7%) had DRP, and of the women without confirmed endometriosis, 5/19 (26.3%) had DRP. Of the 15 women with endometriosis in DRP, 13/15 (86.7%) had Stage II endometriosis and 2/15 (13.3%) had Stage III endometriosis by the r-ASRM classification. Of the women with endometriosis anywhere who completed the postoperative questionnaire, 9/44 (20.5%) took hormonal suppression after surgery. Of the women with deep dyspareunia who completed the postoperative questionnaire, 1/9 (11.1%) of women with DRP and 9/39 (23.1%) of women without DRP (*p* = 0.66) took hormonal suppression after surgery.

A total of 31 DRPs were excised from the 25 women with DRP in the posterior cul-de-sac or uterosacral ligaments, of which 15/31 (48.4%) had endometriosis (Table 1). Ten of the 31 (32.3%) DRPs had no visible lesion on the surface of the pocket. Of these 10 DRPs without visible surface lesions, 3 (30.0%) had endometriosis on histology. The DRP locations where endometriosis most commonly occurred were the left and right uterosacral ligaments, being present in 9/31 (29.0%) DRP for each.

Women with DRP did not differ significantly from women without DRP on age, severe baseline pain symptoms (≥7 for deep dyspareunia, dyschezia, and chronic pelvic pain), or baseline quality of life (Table 2). The two groups did differ significantly in that women without DRP had a higher mean BMI (27.9 ± 7.5 versus 24.6 ± 6.8, *p* < 0.05), higher proportions of Stage I disease (35.8 versus 0.0%, *p* < 0.001) and Stage IV disease (32.1 versus 8.0%, *p* < 0.05), and a lower proportion of Stage II disease (24.7 versus 72.0%, *p* < 0.001) than women with DRP (Table 2).

**Table 2:** **Baseline and surgical characteristics and pain data for 107 women by deep retraction pockets**.

	Retraction pockets^a^ (*N* **=** 25)		No retraction pockets^b^ (*N* **=** 82)		
Characteristic	*n*	%	*n*	%	*p*-value
Age (year) (mean ± SD)	30.5 ± 6.6		31.3 ± 7.0		0.59
Body mass index (kg/m^2^) (mean ± SD)	24.6 ± 6.8		27.9 ± 7.5		<0.05
Obese (body mass index ≥30.0)	4	16.0	28	34.1	0.14
Baseline deep pain with intercourse ≥7	9	40.9	38	59.4	0.21
Baseline pain with bowel movements ≥7	11	61.1	24	49.0	0.55
Baseline chronic pelvic pain ≥7	11	64.7	33	60.0	0.95
Baseline quality of life <70	18	75.0	43	61.4	0.34
ASRM Stage I (minimal) (1–5)	0	0.0	29	35.8	<0.001
ASRM Stage II (mild) (6–15)	18	72.0	20	24.7	<0.001
ASRM Stage III (moderate) (16–40)	5	20.0	6	7.4	0.13
ASRM Stage IV (severe) (>40)	2	8.0	26	32.1	<0.05
ASRM Stage III or IV (moderate or severe) (>16)	7	28.0	32	39.5	0.42

*^a^Baseline deep pain with intercourse ≥7 was unknown for three women who reported baseline deep pain with intercourse; baseline pain with bowel movements ≥7 was unknown for three women who reported baseline pain with bowel movements; baseline chronic pelvic pain ≥7 was unknown for four women who reported baseline chronic pelvic pain; and baseline quality of life was unknown for one woman*.

*^b^Baseline deep pain with intercourse ≥7 was unknown for 18 women who reported baseline deep pain with intercourse; baseline pain with bowel movements ≥7 was unknown for 13 women who reported baseline pain with bowel movements; baseline chronic pelvic pain ≥7 was unknown for 14 women who reported baseline chronic pelvic pain; baseline quality of life was unknown for 12 women; and ASRM stage was unknown for one woman*.

Respondents and non-respondents to the postoperative questionnaire did not differ significantly on age, severe baseline pain symptoms, or baseline quality of life (Table 3). Non-respondents had a significantly higher mean BMI (29.1 ± 8.6 versus 25.1 ± 5.4, *p* < 0.01) and a higher rate of obesity (40.7 versus 18.9%, *p* < 0.05) than respondents.

**Table 3:** **Baseline demographic, pain, and quality of life data for 107 women by postoperative survey**.

	Returned postoperative survey^a^ (*N* **=** 53)		Did not return postoperative survey^b^ (*N* **=** 54)		
Characteristic	*n*	%	*n*	%	*p*-value
Age (year) (mean ± SD)	30.7 ± 6.2		31.6 ± 7.6		0.51
Body mass index (kg/m^2^) (mean ± SD)	25.1 ± 5.4		29.1 ± 8.6		<0.01
Obese (body mass index ≥30.0)	10	18.9	22	40.7	<0.05
Baseline deep pain with intercourse ≥7	21	44.7	26	66.7	0.07
Baseline pain with bowel movements ≥7	17	44.7	18	62.1	0.25
Baseline chronic pelvic pain ≥7	24	61.5	20	60.6	1.00
Baseline quality of life <70	29	61.7	32	68.1	0.67

*^a^Baseline deep pain with intercourse ≥7 was unknown for six women who reported baseline deep pain with intercourse; baseline pain with bowel movements ≥7 was unknown for five women who reported baseline pain with bowel movements; baseline chronic pelvic pain ≥7 was unknown for six women who reported baseline chronic pelvic pain; and baseline quality of life was unknown for six women*.

*^b^Baseline deep pain with intercourse ≥7 was unknown for 15 women who reported baseline deep pain with intercourse; baseline pain with bowel movements ≥7 was unknown for 11 women who reported baseline pain with bowel movements; baseline chronic pelvic pain ≥7 was unknown for 12 women who reported baseline chronic pelvic pain; and baseline quality of life was unknown for seven women*.

Women with DRP and women without DRP both had significant improvement in deep dyspareunia, dyschezia, and chronic pelvic pain after excision surgery (Table 4). Women who had endometriosis in their DRP also had significant improvement in deep dyspareunia and chronic pelvic pain. Quality of life significantly improved after excision surgery, whether or not DRPs were present, and in women who had endometriosis in their DRP. All significant improvements in pain symptoms and quality of life remained after women who reported hormonal suppression after surgery were removed from the analyses (data not shown).

**Table 4:** **Pre/postoperative pain and quality of life data for 107 women by deep retraction pockets**.^f^

Characteristic	*N*	Preoperative score^c^ (median)	Postoperative score^d^ (median)	Difference score^e^ (post–pre) (median)	*p*-value
**Deep pain with intercourse score**^a^					
Deep retraction pockets	13	6.0	4.0	–2.0	<0.01
Endometriosis	8	5.0	0.0	–3.0	<0.05
No endometriosis	5	8.0	8.0	–1.0	0.50
No deep retraction pockets	32	6.0	4.0	–2.0	<0.001
**Pain with bowel movements score**^a^					
Deep retraction pockets	11	7.0	0.0	–6.0	<0.05
Endometriosis	7	7.0	0.0	–6.0	0.06
No endometriosis	4	7.0	2.0	–5.5	0.31
No deep retraction pockets	25	6.0	0.0	–4.0	<0.001
**Chronic pelvic pain score**^a^					
Deep retraction pockets	11	7.0	0.0	–3.0	<0.01
Endometriosis	7	7.0	0.0	–3.0	<0.05
No endometriosis	4	6.5	4.0	–2.5	0.28
No deep retraction pockets	26	7.0	0.0	–4.0	<0.001
**Quality of life score**^b^					
Deep retraction pockets	12	55.0	80.0	25.0	<0.05
Endometriosis	7	50.0	90.0	30.0	<0.01
No endometriosis	5	60.0	70.0	15.0	0.53
No deep retraction pockets	33	50.0	80.0	25.0	<0.001

*^a^Symptoms were self-reported on a 10-point scale of increasing severity from 1 (mild) to 10 (severe) on the preoperative questionnaire and from 0 (none) to 10 (severe) on the postoperative questionnaire indicating improvement/worsening of symptom or complete elimination of symptom*.

*^b^Symptoms were self-reported on a 100-point scale from 0 (worst) to 100 (best)*.

*^c^Preoperative scores did not differ significantly between deep retraction pockets and no deep retraction pockets groups (deep pain with intercourse: *p* = 0.94, pain with bowel movements: *p* = 0.71, chronic pelvic pain: *p* = 0.94, and quality of life: *p* = 0.86)*.

*^d^Postoperative scores did not differ significantly between deep retraction pockets and no deep retraction pockets groups (deep pain with intercourse: *p* = 0.65, pain with bowel movements: *p* = 1.00, chronic pelvic pain: *p* = 1.00, and quality of life: *p* = 0.64)*.

*^e^Difference scores did not differ significantly between deep retraction pockets and no deep retraction pockets groups (deep pain with intercourse: *p* = 0.95, pain with bowel movements: *p* = 0.89, chronic pelvic pain: *p* = 0.55, and quality of life: *p* = 0.67)*.

*^f^There were no differences in the statistical significances of the analyses performed with the 10 patients removed who took hormonal suppression postoperatively*.

## Discussion

This study confirms the association of DRP, also called Allen-Masterson windows (2), and endometriosis. In our study, 25/107 (23.4%) of the women with deep dyspareunia had DRP within the posterior cul-de-sac or uterosacral ligaments, the majority of whom (15/25, 60%) had endometriosis diagnosed by histology in the pocket [Vilos (8) and Moen (9), only 12%]. These results are comparable to those of other studies looking at the association of retraction pockets and endometriosis, including Chatman (3) (17%) and Redwine (4) (18%). Our study adds the observation that even a clear pocket, a DRP without a visible surface lesion inside the pocket, can be associated with endometriosis up to 3/10 (30%) of the time. Thus, the retraction pocket itself, with or without visible surface lesions, can be a manifestation of endometriosis and should be treated.

The origin of retraction pockets has been postulated to result from peritoneal irritation or invasion by endometriosis, with resultant scarring and retraction of the peritoneum (2, 3). It also has been suggested that a retraction pocket may be a cause of endometriosis, where the disease presumably settles in a previously altered peritoneal surface (10). Since 16/31 (51.6%) of DRP did not have endometriosis in our study, it seems more likely that retraction pockets represent a primary developmental formation defect of the pelvic peritoneum. Since the rate of DRP was similar in patients with endometriosis 20/88 (22.7%) and patients without endometriosis 5/19 (26.3%), our study also does not support the postulate that endometriosis causes DRP (4). It is possible that endometriosis and DRP are unrelated in their origins.

Since endometriosis can be present (by histology) even if the surface of a DRP is clear, and given that quality of life improves when all manifestations of endometriosis are excised, including DRP, we recommend that DRP be removed at the time of excision surgery in patients with deep dyspareunia. Our patients had significantly improved quality of life after excision surgery, whether or not DRPs were present. Given that this study demonstrated that a DRP may be a manifestation of endometriosis (with or without visible lesions on the surface of the pocket), DRP also should be excised since the rate of recurrence or persistence of endometriosis has been shown to be very low after optimal excision of all possible visible manifestations of endometriosis (11). Since DRPs always were excised when present, further studies comparing outcomes after excision of DRP versus not excising DRP are needed to confirm this recommendation.

Limitations of our study include the lack of complete follow-up data (49.5% completion rate), lack of validated sexual functioning scales in measuring patient outcomes, lack of more detailed quality of life measures, and the lack of a comparison group where DRPs were seen but not treated. Strengths of our study include the prospective data collection of symptoms, quality of life before and after surgery and of the operative data, and consistency in patient treatment by a single surgeon committed to optimal excision.

Medical therapy has been shown to improve deep dyspareunia and quality of sex life (12). The majority of patients in our study did not take postoperative hormonal suppression. Future research also should compare medical and surgical therapies for deep dyspareunia and quality of life. Again, we recommend that validated sexual functioning questionnaires be used to assess patient outcomes in such studies.

## Ethics Statement

The authors declare that the research was conducted in compliance with ethical standards.

## Author Contributions

PY: project development, manuscript writing, and final approval of manuscript. IL: data collection and data management. JG: data management, data analysis, manuscript writing, and final approval of manuscript.

## Conflict of Interest Statement

The authors declare that the research was conducted in the absence of any commercial or financial relationships that could be construed as a potential conflict of interest.
